# MSPEECH (multiple sclerosis monitoring through speech interaction in clinic and at home): a Living Lab study protocol for co-created, speech-based digital biomarkers in multiple sclerosis

**DOI:** 10.3389/fdgth.2026.1830655

**Published:** 2026-07-16

**Authors:** Tina Lemkau, Hernan Inojosa, Anja Dillenseger, Johannes Tröger, Janna Hermann, Nicklas Linz, Stephen Gilbert, Tjalf Ziemssen

**Affiliations:** 1 Center of Clinical Neuroscience, Department of Neurology, Faculty of Medicine and University Hospital Carl Gustav Carus, TUD Dresden University of Technology, Dresden, Germany; 2 Centre for Tactile Internet with Human-in-the-Loop (CeTI), TUD Dresden University of Technology, Dresden, Germany; 3 Ki:Elements GmbH, Saarbrücken, Germany; 4 Else Kröner Fresenius Center for Digital Health, TUD Dresden University of Technology, Dresden, Germany

**Keywords:** artificial intelligence, digital tools adoption, Living Lab (LL), multiple scleorsis (MS), patient—centered design, speech—brain

## Abstract

Multiple sclerosis (MS) has heterogeneous trajectories and fluctuating symptoms that are often not captured by intermittent clinic assessments. Digital health tools can provide frequent, low-burden measurements, but clinical translation requires sustained engagement, interpretability, robust governance, and workflow integration. MSPEECH is a single-center, prospective, non-interventional Living Lab study at the MS Center Dresden conducted with ki:elements GmbH to co-create and iteratively refine the Mili smartphone/tablet speech assessment app. The protocol comprises three demonstrator cycles with structured co-design (people with MS and clinicians), implementation within predefined boundaries while keeping core tasks/schedules/security stable, and a 12-week home-based evaluation per version. The battery includes multiple speech tasks (e.g., fluency, reading, “Pa–Ta–Ka,” sustained phonation) and a dual-task module combining touchscreen fine-motor tracking with concurrent speech. A dedicated Ethical, Legal and Social Issues (ELSI) board provides continuous oversight. Primary outcomes are usability and acceptability (MAUQ plus qualitative feedback); secondary outcomes include feasibility/engagement, privacy perceptions, report interpretability, clinician workflow fit, and exploratory associations with clinical reference indicators.

## Introduction

Multiple sclerosis (MS) is a chronic inflammatory disease of the central nervous system with heterogeneous trajectories, fluctuating symptom burden, and multi-domain disability (1). Contemporary diagnostic and phenotyping frameworks emphasize the need to capture disease activity and progression across relapsing and progressive phenotypes over time, yet routine clinical workflows still rely heavily on intermittently collected in-clinic measures and patient reports (2, 3). This approach can miss short-term variability and underestimate subtle functional decline (4). In practice, gait and mobility are often the most consistently assessed functional domains, while other prevalent and impactful clinically relevant MS symptoms (e.g., cognition, fatigue, speech, and mood) may be assessed less systematically and therefore risk being overlooked (5).

Digital health approaches provide more frequent, low-burden measurements in everyday environments (6). In MS, smartphone-based “active tests” can support remote monitoring of function, capture symptom dynamics between visits, and complement standard clinical outcomes, supporting a shift toward digital endpoints and biomarkers (7, 8). However, the development of clinically relevant digital measures remains challenging: many tools show early feasibility but fail to achieve sustained engagement, interpretability, or integration into routine care, while accounting for legal and ethical constraints (9). Real-world sustainable development of digital biomarkers therefore requires structured co-creation, documentation, and risk control—principally once a system is positioned toward clinical use (10).

Speech is a particularly attractive candidate for digital biomarker development in MS (11–15). Speech production and language reflect a combination of neurologic domains, including motor control, respiratory and bulbar function, cognitive processing, and affective factors, that can be affected early and throughout the disease related to neuroinflammation, demyelination, or cognitive load (11, 13, 16–18). Importantly, speech tasks are scalable, require minimal equipment, and can be repeated frequently without clinic infrastructure (e.g., through smartphones/tablets) (14, 19–21). Recent work supports the feasibility of automatic extraction of acoustic and linguistic speech metrics in MS and suggests that speech metrics may align with symptom severity and clinically relevant domains (11, 22). Moreover, fatigue-related changes may be expressed in speech tempo, pause structure, and acoustic variability, making speech a potential candidate marker for day-to-day symptom dynamics (22).

Despite this potential, the translation of speech into clinically useful tools requires more than demonstrating correlations. It requires a development pathway that makes speech capture acceptable, secure, interpretable, and clinically useful. This includes iterative usability refinement, evaluation of engagement and adherence, careful handling of privacy risks inherent to voice data, and early consideration of regulatory and clinical integration requirements. In this context, Living Lab approaches as real-world, stakeholder-inclusive, iterative co-creation ecosystems have been proposed as a mechanism to bridge the step from technical feasibility to clinical usefulness (10, 23).

Adopting principles of Living Labs tailored to digital biomarker development, MSPEECH implements a regulation-aware methodology to address this translational gap as the first MS Living Lab (10). The project aims to develop, refine, and evaluate a speech assessment app (Mili) across successive demonstrator versions, integrating input from people with MS (pwMS), clinicians, and app developers. It combines structured co-creation cycles, explicit ethical and legal governance, secure data handling, and a focus on meaningful integration into care pathways.

In parallel, MSPEECH builds on and connects to existing collaborations and prior speech-related project work (including earlier app-based speech studies and collaboration with the technology partner ki:elements GmbH), while extending the methodological focus to implementation readiness and governance. Here, we outline the MSPEECH development protocol, including co-creation cycles, governance, usability evaluation, and an exploratory analysis plan across three demonstrator iterations, rather than a clinical efficacy study or final digital biomarker validation.

## Protocol

The sections below describe the structured Living Lab cycles, recruitment, procedures, outcomes, governance, and the planned exploratory analyses for the MSPEECH development phase.

## Study design and Living Lab translation

**Figure 1:**
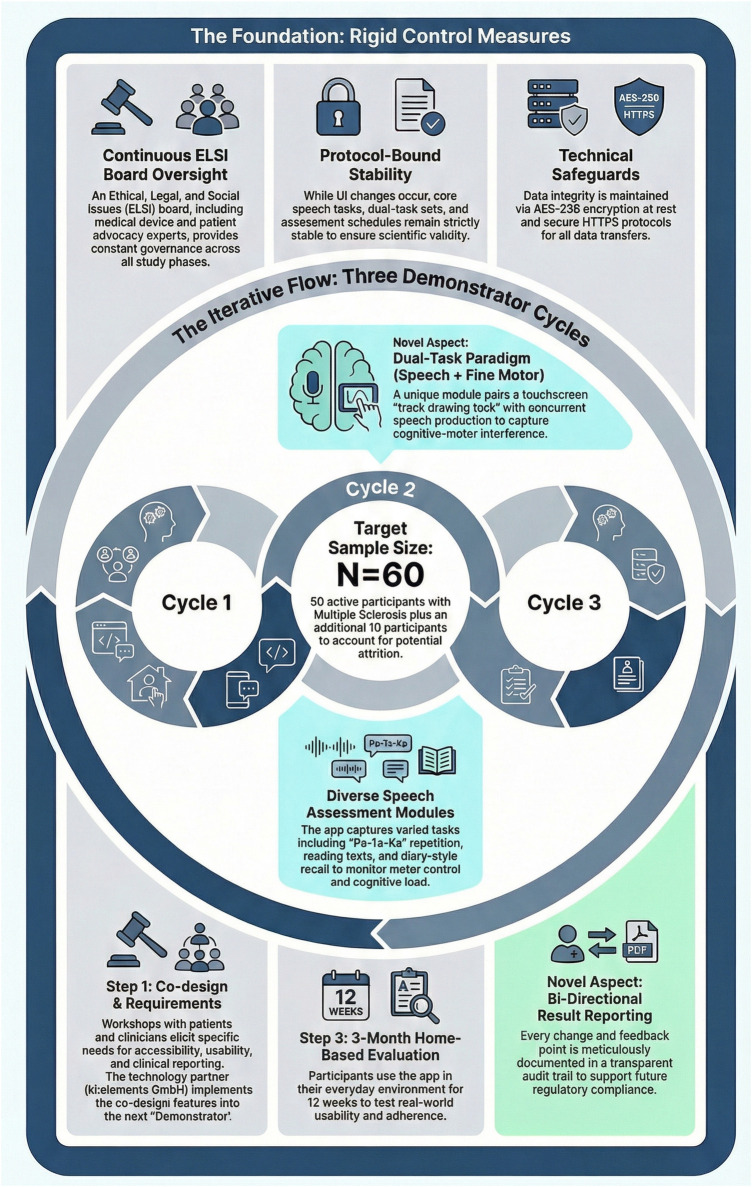
Regulation-aware Living Lab framework and iterative demonstrator cycles of the MSPEECH/Mili speech assessment app. The upper panel summarizes the fixed control measures applied across all iterations: continuous oversight by an Ethical, Legal and Social Issues (ELSI) board, protocol-bound stability of the core assessment content (speech tasks, dual-task module, and assessment schedule) despite user-interface (UI) refinements, and technical safeguards for secure data handling (encryption at rest, e.g., AES-256, and encrypted transfer via HTTPS). The central panel illustrates the iterative flow across three sequential demonstrator cycles, each comprising stakeholder co-design (people with multiple sclerosis and clinicians), implementation by the technology partner, and real-world evaluation. The target sample size for the development phase is *N* = 60 (50 active participants plus ∼10 to offset attrition). The assessment battery includes diverse speech modules (e.g., diadochokinesis “Pa–Ta–Ka”, Reading, free speech/recall) and a novel dual-task paradigm combining touchscreen fine-motor tracking with concurrent speech to probe cognitive–motor interference. The lower panel highlights key operational steps, including co-design workshops, a 12-week home-based evaluation period per demonstrator, and bi-directional result reporting (participant PDF feedback and clinician-facing outputs) with documented version control to support auditability and future regulatory readiness.

MSPEECH is a single-center, prospective, non-interventional Living Lab study conducted at the Center for Clinical Neurosciences/MS Center Dresden (ZKN), University Hospital Carl Gustav Carus Dresden, in collaboration with ki:elements GmbH (technology partner and owner of the Mili app). The study is structured as an iterative development and evaluation protocol, with approximately three co-creation demonstrator versions evaluated in sequence, involving all stakeholders (including pwMS, treating clinicians, technology partners and regulatory staff). An iteration cycle includes: (1) co-design feedback and requirement elicitation with pwMS and clinicians; (2) implementation by the technology partner; (3) usability and acceptability evaluation with end users; and (4) documentation of changes, decisions, and risk considerations (Figure 1).

Progression from one demonstrator cycle to the next is based on structured multi-stakeholder evaluation including usability outcomes, qualitative participant feedback, and clinician evaluation. Demonstrator refinement and progression decisions require that no major unresolved usability, accessibility, ethical, legal, privacy-related, or patient-safety concerns remain. Given the exploratory co-creation design, no single quantitative progression threshold was predefined; decisions are consensus-based and documented within the governance framework.

The Living Lab logic is implemented through an explicitly structured, protocol-bound iteration process (10). Across cycles, the core task set, assessment schedule windows, and the security baseline remain unchanged; iteration is limited to defined user interface elements, onboarding/access pathways, and reporting formats within pre-specified boundaries. Consistent with the regulation-aware Living Lab roadmap, MSPEECH treats iteration as a managed, documented activity rather than *ad hoc* change. This structure enables improvement of usability and reporting while maintaining a stable investigative purpose (development and evaluation of UI/usability and integration into MS management) and a transparent audit trail of changes.

A distinctive feature is the integration of an Ethical, Legal, and Social Issues (ELSI) board into the Living Lab governance (24). The protocol defines ELSI oversight for data protection, data security, legal and ethical aspects, and patient interest representation. The ELSI board consists of two members with complementary expertise: one member with expertise in ethics, regulatory science, and medical device governance, and one patient representative with extensive experience in patient advocacy, patient safety, and user perspectives in digital health contexts. The members were selected based on their subject-matter expertise and their ability to provide independent ethical and patient-centred perspectives throughout the project. Neither ELSI board member maintains a financial relationship with ki:elements GmbH.

The ELSI board is regularly consulted during the development of study materials, questionnaires, participant information, and other project-related procedures to ensure consideration of ethical, legal, social, usability, and patient safety aspects. The board has an advisory role and is not involved in the operational conduct of the study or in primary scientific decision-making. Given the two-member composition, quorum requires participation of both members. Recommendations are developed through consensus-based discussion. If a conflict of interest emerges, the affected member is recused from the relevant discussion and the matter is escalated to the project leadership and, where necessary, to the wider consortium or relevant institutional structures. Ethical or patient-safety-related concerns identified by the board are escalated to the project leadership and discussed within the project consortium.

This governance element aims to translate Living Lab co-creation into a form compatible with medical context requirements: stakeholder inclusion without sacrificing accountability, documentation, or trust.

## Setting

The study is coordinated and clinically anchored at the ZKN. Recruitment and participant support are provided by the study team. Furthermore, clinicians at ZKN participate in the process of co-creation and evaluation of reporting formats and integration into clinical workflows.

## Participants: selection, inclusion, exclusion

PwMS are eligible if they are adults (≥18 years), have MS according to the 2017 McDonald criteria, provide written informed consent, have sufficient German proficiency to understand the instructions and provide samples, and have a compatible smartphone or tablet capable of running the app. Only persons able to provide consent are included; if motor impairment prevents signing, consent can be documented via witnessed confirmation. Exclusion criteria are minority age, inability to consent, or clinical judgement that health limitations prevent participation. Treating physicians at ZKN are eligible to participate as clinician end users for the evaluation of the integration and visualisation and the scope of reported results into clinical routine and the patient file. The protocol prioritizes heterogeneity: participants are included unselected and in order of presentation/registration to represent different MS phenotypes, ages, and symptom profiles, supporting generalizability of usability findings across the MS spectrum.

## Recruitment and enrolment procedures

The call for participation is disseminated through patient portals, clinical contact lists, and in-clinic information channels at ZKN; interested participants contact the study team for information and enrolment. Informed consent is obtained prior to any data collection. Participants can choose between participation in both development (co-creation workshops) and evaluation, or to evaluation only.

## Intervention/exposure and procedures to be measured

MSPEECH is non-interventional in the clinical sense; the “intervention” is exposure to successive versions of the speech demonstrator UI/workflows and the performance of structured speech and dual-task assessments in the app. Data collection is active only (no passive audio capture), occurring exclusively during user interaction with the app. Audio is recorded only during explicitly initiated task modules.

## Speech assessment modules

The protocol includes several classes of speech tasks designed to investigate complementary domains relevant to MS symptomatology and functional change (11, 22): picture description (free speech production), verbal fluency (phonemic and semantic), a diary-style recall narration (free speech plus memory retrieval load), reading texts (controlled speech production under standardized linguistic input), diadochokinetic “Pa-Ta-Ka” repetition (articulatory motor control), and sustained phonation (voice/prosody source characteristics). Speech recordings are stored as WAV files and processed to extract exploratory acoustic, temporal, articulatory, prosodic, fluency-related, and linguistic measures. These may include speech rate, pause structure, articulation timing, phonation stability, pitch and intensity variability, lexical diversity, verbal fluency metrics, and dual-task-related speech changes. These tasks were selected to support both usability-driven refinement (are tasks understandable, acceptable, feasible at home?) and exploratory analytic objectives (can extracted features provide stable, interpretable digital measures across repetitions and user subgroups?). The protocol explicitly notes the intention to enable future objective quantification of cognition, depression, fatigue, fine motor function, and speech functionality via the final demonstrator. Further methodological specifications are provided in Supplementary Table 1.

## Dual-task paradigm (speech + fine motor)

A dual-task module combines a fine motor “track drawing task” (TDT) on the touchscreen with concurrent speech production tasks, including semantic fluency (patients for example must recall as many words of a category, e.g., animals, as possible) and rapid “Pa-Ta-Ka” sequences. Each fine-motor dual-task trial lasts one minute; a one-minute single-task TDT control trial is recorded at the end, and brief familiarization trials precede data acquisition. The dual-task component is intended to capture speech performance under added motor load and to explore a home-based paradigm that may reflect cognitive-motor interference relevant to MS functional impairment.

## Schedule and study flow

The development process consists of approximately three workshops with pwMS and clinicians, followed by implementation and release of successive demonstrator versions. Workshops are led by ZKN with involvement of the ELSI board. In the first workshop, requirements for the speech analysis system and evaluation materials are defined, incorporating (i) usability and symptom-related accessibility needs (e.g., contrast settings, interaction constraints), and (ii) ethical and legal requirements. For each deployed demonstrator version, participants test the app from home over approximately three months. Tasks are performed weekly during the first four weeks and then monthly thereafter; each session lasts approximately 10–15 min. Participants are instructed to perform assessments in a quiet room without interruptions; instructions are delivered visually (including short videos) within the app. After each test phase, iterative evaluation is conducted via interviews, workshops and surveys. Importantly, evaluation includes both returning participants and “fresh” participants who did not define requirements, to assess intuitive use and reduce bias from prior exposure. Results of each workshop are prepared by ZKN and transferred as anonymous reports to ki:elements GmbH to guide subsequent demonstrator refinements.

## Outcomes

Because MSPEECH is a Living Lab development and evaluation protocol, outcomes include both implementation-focused endpoints (usability, acceptability, feasibility, ELSI dimensions, integration into clinical workflow) and exploratory analytic endpoints (speech metric performance relative to clinical reference data) (25). The protocol specifies that evaluation occurs on two levels: qualitative evaluation of usability/acceptance/ELSI aspects and quantitative evaluation of effectiveness/performance measured against clinical reference data, plus evaluation of clinical integration and integration of speech results into MS therapy management with clinicians. To operationalize these aims, MSPEECH defines the following primary and secondary outcomes for the present protocol phase.

Primary outcome: the usability and acceptability of each demonstrator version, assessed via mixed methods combining standardized usability questionnaires and qualitative user feedback (interviews/workshops), focusing on task comprehension, interaction burden, perceived value, and trust-related dimensions. A validated app usability instrument (interactive version of the Mobile App Usability Questionnaire, MAUQ) will be used to quantify usability alongside qualitative thematic analysis (26, 27). Given the exploratory and iterative Living Lab design, no predefined minimal clinically important difference (MCID) threshold is specified; usability changes will therefore be interpreted descriptively and in conjunction with qualitative stakeholder feedback.

Secondary outcomes: feasibility and engagement metrics (completion rates, session duration, longitudinal adherence across the 3-month phase, reasons for non-adherence and drop-out); user-experience and accessibility needs stratified by symptom profiles and age groups; perceived appropriateness and clarity of the PDF report and clinician-facing display; ELSI-relevant outcomes including perceived privacy, data control, and acceptability of remote speech capture; and exploratory associations between extracted speech/dual-task measures and available clinical reference indicators from routine care, including disability-related measures such as the Expanded Disability Status Scale (EDSS), symptom questionnaires, and patient-reported outcomes, interpreted as hypothesis-generating.

For clarity, usability in the present protocol refers to the perceived ease of learning, interaction, navigation, task execution, and understandability of the demonstrator application in home-based settings. Acceptability refers to the perceived appropriateness, burden, trustworthiness, and willingness for continued use of the system. Feasibility refers to practical implementation characteristics including adherence, completion rates, and sustained engagement over repeated home-based assessments.

## Sample size and sample size rationale

We estimate that approximately 50 PwMS are required for the development and evaluation of Demonstrator 1 and 2, with an additional ∼10 participants enrolled to replace potential drop-outs, yielding a target sample of approximately 60 for the present development/evaluation phase. This sample size is aligned with usability and Living Lab development work, where mixed-methods feedback saturation and robust estimation of usability scores typically occur in samples of this magnitude.

The pragmatic statistical rationale is as follows. For standardized usability scales scored on a 1–7 or 1–5 Likert scales, *n* ≈ 50–60 allows estimation of mean usability with reasonably tight confidence intervals. Assuming a standard deviation of 0.9 points (typical in app usability studies), the 95% CI half-width for the mean is approximately 1.96 × 0.9/√60 ≈ 0.23 points, enabling discrimination between “acceptable,” “good,” and “excellent” usability bands at the group level. For within-participant comparisons between successive demonstrator versions (paired designs), *n* ≈ 52 achieves ∼80% power to detect a moderate standardized mean difference of 0.4 at *α* = 0.05, supporting detection of practically meaningful UI improvements across iterations. In line with feasibility methodology, the goal is not hypothesis confirmation but learning, optimization, and evidence sufficiency to justify subsequent, larger confirmatory studies (28). The protocol also anticipates a follow-up project to evaluate the final demonstrator's usability and its integration into MS monitoring by clinicians; funding for that stage is not yet secured.

## Data sources, data handling, and security

Data types include app-captured audio recordings (WAV), participant ID, and selected demographic variables including age and years of education (the latter is required for interpreting certain test outputs), plus questionnaires and interview data regarding the MSPEECH system and demonstrator versions. Identifying information (name, contact details, consent date) is stored separately on password-protected university hospital servers with restricted access.

Speech data are collected via Mili on iOS/Android devices. Data are only captured during app usage; there is no passive data collection. Recordings are encrypted and temporarily stored on the device until upload is possible and then deleted from the device after successful transfer. Data transfer uses encrypted protocols (HTTPS). Server infrastructure includes network security and controlled access. The speech demonstrator is hosted in a Kubernetes cluster in a data center located in Frankfurt/Main, Germany; stored data (database tables, temporary files, backups) are encrypted with AES-256 or better. A key translational mechanism will be the return of results: test outputs will be returned to participants as a PDF via the same secure pathway, and the ZKN will receive outputs for planned integration into the hospital's patient record system. The protocol specifies that the technology partner accesses only university hospital servers remotely via secure SSH/SSL with strong authentication methods. This bidirectional reporting is central to the MS Living Lab translation: it supports perceived participant value (potentially improving engagement) while allowing clinicians to evaluate interpretability and workflow compatibility early, before any claims of clinical decision support are made.

## Statistical analysis plan

Analyses are explicitly exploratory for this Living Lab phase. The protocol describes descriptive statistics for interval-scale variables (including missingness, percentiles, mean, SD) and distributions for ordinal/nominal variables. For comparisons across conditions and groups, analyses will remain exploratory. Descriptive summaries will be reported per demonstrator version and over time. Where repeated measures are available, mixed-effects models will be used to estimate changes across demonstrator iterations and within-person trajectories; supportive paired comparisons may be reported. Effect estimates with confidence intervals will be emphasized; *p*-values will be interpreted descriptively without multiplicity adjustment.

To account for the iterative co-creation structure of the study, analyses will distinguish between returning participants exposed to previous demonstrator versions and first-time participants entering a given cycle without prior exposure. Mixed-effects models may therefore include participant type (returning vs. newly enrolled participant) as an exploratory covariate where appropriate. Participant-specific random effects will be used to account for repeated measurements within individuals.

Exploratory mixed-effects analyses may additionally include relevant covariates such as age, education level, disability severity, symptom burden, and participant type where appropriate and data availability permits.

Additional exploratory analyses may assess potential carryover, learning, or familiarity effects associated with repeated exposure to prior demonstrator versions, particularly regarding usability ratings, task completion behaviour, and interaction patterns.

For the present protocol, the statistical plan can be expanded to align with the dual evaluation levels. Qualitative data (interviews, focus groups, free-text feedback) will undergo thematic analysis with coding guided by Living Lab objectives: usability barriers/facilitators, accessibility needs, trust and privacy perceptions, perceived clinical value, and recommendations for report design and task flow. Quantitative usability outcomes will be summarized per demonstrator version and compared within participants when applicable. Engagement outcomes (adherence, completion rates) will be analysed longitudinally, including attrition patterns (9). Exploratory modelling of speech metrics may include feature stability (test–retest reliability proxies across repeated sessions), association with symptom questionnaires, and subgroup analyses (age, phenotype proxies, disability severity), recognizing that effect estimates will be interpreted cautiously and used to prioritize features for subsequent validation studies.

The repeated home-based assessments across demonstrator phases additionally allow exploratory evaluation of within-participant stability and test–retest characteristics for selected speech and dual-task measures. Where appropriate, exploratory reliability analyses (e.g., intraclass correlation coefficients) may be performed for repeated measures collected under comparable conditions.

## Ethical considerations and approvals

Participation in the study is voluntary, requires written informed consent, and can be withdrawn at any time without consequences for care. Upon withdrawal, all identifiable data are deleted; data already included in completed analyses cannot be removed. The study is conducted in full compliance with the ethical principles for medical research involving human subjects, as outlined in the Declaration of Helsinki (29). The legal framework for data protection is defined by the EU General Data Protection Regulation (GDPR) and the German Federal Data Protection Act (Bundesdatenschutzgesetz, BDSG). All procedures for data collection, storage, and processing adhere strictly to these regulations. Given that the Mili app is currently an investigational tool and not a certified medical device, the study is classified as non-interventional research. However, the protocol's “regulation-aware” design anticipates the potential future classification of the tool under the EU Medical Device Regulation (MDR), ensuring that documentation and governance practices are aligned with future regulatory requirements.

The protocol and all relevant documents (including consent forms and participant information sheets) were approved by the responsible ethics committee at the Technical University of Dresden [BO ff (Mono)-EK-220052025]. The integrated ELSI board provides an additional layer of continuous oversight, monitoring privacy, security, and ethical considerations throughout the iterative development process, which is particularly crucial for handling inherently identifiable speech data.

## Dissemination and data sharing plan

The protocol's dissemination strategy includes the development of a final speech demonstrator optimized for end-user needs and integration pathways, with subsequent projects planned to evaluate the final demonstrator's usability and clinical integration more broadly.

For scholarly dissemination, the reporting of study findings will be guided by established international standards to ensure transparency and reproducibility. While the present study is not a conventional clinical trial, the principles of structured reporting for both the protocol and the observational results will be applied to improve the clarity and reusability of the findings.

The study will disseminate outcomes through peer-reviewed publications and conference presentations, with a focus on (i) the translation of Living Lab methodology into regulated digital health contexts, (ii) usability and engagement findings, (iii) data governance and trust mechanisms for speech data, and (iv) the exploratory performance of speech-derived measures in relation to clinical reference data.

Regarding associated data, manuscripts will specify what derived, de-identified features (rather than raw audio) can be shared under GDPR-compatible governance and participant consent, and under what access controls. Where feasible, sharing of analysis code is encouraged to support reproducibility and external validation, acknowledging that raw speech data require stricter governance due to re-identification risk.

## Study risk assessment

The study risk assessment encompasses the systematic identification, analysis, and control of risks to participant safety, data integrity, and scientific validity. The iterative nature of the Living Lab methodology fundamentally shapes this process, transforming risk management from a static, pre-study activity into a dynamic, continuous function integrated throughout the study's lifecycle. The iterative design introduces a unique risk-benefit trade-off. On one hand, each modification cycle such as adjustments to the user interface, feedback reports, or onboarding workflows carries the potential to introduce new risks. These may include software bugs, usability challenges that could lead to participant frustration or data errors, and emergent privacy concerns as the system's functionality evolves.

On the other hand, the iterative approach is the core mechanism for risk mitigation. By developing and evaluating the digital biomarker tool in sequential, protocol-bound cycles, the study contains the impact of any potential issue. Risks are identified within a small, controlled group of participants during a specific demonstrator phase, allowing them to be resolved before they can affect the broader cohort or compromise the study's overall objectives. This “fail-fast, learn-early” paradigm systematically de-risks the technology and its implementation, ensuring that each successive version is more robust, secure, and user-friendly.

## Explicit risk control mechanisms

Risk is explicitly controlled through a multi-layered governance and operational framework that remains consistent across all iterations:

1. Protocol-Bound Iteration: The study protocol strictly defines the scope of permissible changes within each cycle. Core scientific and security components, such as the primary speech and dual-task sets, assessment schedules, and the foundational security architecture, remain stable. Iteration is confined to pre-specified, non-core elements, and is treated as a “managed, documented activity” with transparent version control, rather than an ad-hoc process. This ensures that the study's scientific integrity and safety baseline are never compromised.
2. ELSI Governance: A dedicated Ethical, Legal, and Social Issues (ELSI) board provides continuous oversight across all iterations. This board includes experts in medical device regulation and patient advocacy and is tasked with prospectively and retrospectively evaluating risks related to data privacy, security, and participant interests. Given the inherently identifiable nature of speech data, this governance layer serves as a critical, ongoing mechanism for risk control that extends beyond initial ethics committee approval.
3. Structured Co-Creation and Evaluation: Each iteration cycle includes mandatory co-design workshops and formal usability and acceptability evaluations with end-users (pwMS and clinicians). This structured feedback loop ensures that risks related to human-computer interaction, task comprehension, and perceived value are identified and addressed systematically.
4. Transparent Documentation: All changes, decisions, and risk considerations are meticulously documented, creating a “transparent audit trail.” This documentation provides a clear and auditable history of the system's evolution and the rationale for each modification, ensuring accountability and supporting potential future regulatory submissions.
5. Technical Security Controls: The protocol specifies a robust and non-negotiable security architecture. This includes end-to-end data encryption during transfer (HTTPS), encryption of data at rest (AES-256 or better), secure data hosting in a GDPR-compliant data center, and strictly controlled, authenticated access protocols. These technical safeguards provide a stable foundation for data protection that persists through all iterative changes to the application.

Together, these mechanisms ensure that while the MSPEECH study embraces the flexibility of iterative development, it does so within a rigid framework of risk control that prioritizes participant safety, data security, and scientific validity.

## Conclusions/discussion

MSPEECH operationalizes a regulation-aware Living Lab approach for digital biomarker development in MS by combining iterative co-creation with structured governance, documentation discipline, and explicit attention to data security and clinical integration (30). The protocol advances beyond “app feasibility” by treating usability, adherence, and trust as first-class scientific targets, not merely implementation details. This positioning is strongly aligned with broader lessons from digital biomarker programs: technical feasibility does not guarantee real-world utility, and sustained engagement depends on perceived value, low burden, and credible governance (25).

A central methodological contribution is the translation of Living Lab principles into an auditable, protocolized iteration cycle suitable for digital health systems moving toward clinical use (23, 31). Our published roadmap stresses that Living Labs in health must not equate iteration with uncontrolled change; rather, iteration should occur within pre-defined boundaries, supported by stakeholder governance and continuous risk assessment (10). MSPEECH translates this by structuring three demonstrator cycles, each anchored by workshops/interviews, real-world deployment, and formal feedback loops to the technology partner ki:elements GmbH. This creates a change narrative that can later support regulatory evidence building if the tool evolves from demonstrator to medical device, while still enabling rapid learning in early phases.

The protocol's ELSI board and explicit security architecture are particularly salient for speech. Speech is simultaneously low-burden and highly sensitive: it is easy to collect but can reveal identity, emotion, health status, and social background. Embedding ELSI into the Living Lab process—rather than limiting ethics to initial committee approval—creates a mechanism to manage emergent concerns (e.g., participant comfort with certain tasks, preferences about result feedback, or perceived surveillance) and to align with societal expectations about privacy and fairness. This governance model may be transferable to other neurological digital biomarker domains that rely on inherently identifiable signals (voice, video, free text).

MSPEECH also emphasizes early clinical integration. Returning results as PDFs to participants and enabling clinician access through secure hospital servers supports two Living Lab goals: co-creation of meaningful outputs and evaluation of workflow fit. This is strategically important because many digital biomarker projects stall at the “measure” stage; clinicians may not adopt outputs unless they are interpretable, concise, and compatible with time constraints. Co-designing the report layout and content with clinicians, while simultaneously capturing patient preferences for feedback, may reduce downstream implementation barriers. However, participant-facing PDF reports are intended for exploratory and informational purposes only and are not designed to provide diagnostic or therapeutic recommendations. Accompanying communication explicitly emphasizes the investigational status of the extracted speech-related measures and clarifies that the presented outputs are not validated clinical decision-support metrics.

From a measurement perspective, MSPEECH intentionally spans multiple speech task types (free speech, controlled reading, articulatory and phonatory tasks) and adds a dual-task module that pairs fine motor control with speech production. This breadth acknowledges that MS manifestations are heterogeneous and that different tasks may be sensitive to different domains (motor speech, executive control, fatigue, affect). Prior work suggests that speech metrics can reflect MS-related impairment and may serve as digital biomarkers when extracted automatically and analysed systematically (11, 22). The present protocol appropriately frames analytic findings as exploratory; a key output will be the identification of features that are stable enough for longitudinal tracking and sufficiently robust across devices and contexts. Lessons from other MS smartphone programs—including device heterogeneity and measurement equivalence—reinforce why this robustness work is non-negotiable in real-world deployment (32–34).

The sample size and analysis choices reflect the development nature of the study. A cohort of ∼60 participants is unlikely to support definitive clinical endpoint claims but is well suited to (i) detect usability improvements across iterations, (ii) characterize adherence and attrition patterns, and (iii) generate effect-size estimates and feature prioritizations needed for powered confirmatory studies. This staged evidence approach is consistent with the logic of complex intervention development and digital health evaluation, where early phases focus on optimization and feasibility before scaling.

Several limitations are inherent. First, monocentric recruitment may influence generalizability of usability findings due to local care pathways, participant demographics, and centre-specific digital literacy support. To mitigate this, in addition to patients of the ZKN, pwMS who are treated elsewhere can also participate in the study. Second, restricting inclusion to native German speakers simplifies language processing but limits immediate transferability to multilingual MS populations; however, it also reduces confounding in early feature development. Third, result feedback may increase engagement but also introduces the risk of misinterpretation (participants may over-attribute meaning to exploratory metrics). The protocol mitigates this by positioning outputs as part of development and clinician evaluation, and by embedding ELSI oversight. Future expansions should include explicit communication strategies and user education to prevent harm from overinterpretation.

In summary, MSPEECH proposes a concrete template for implementing Living Lab innovation in a way that remains compatible with the expectations of clinical research and, potentially, future regulatory pathways. By integrating co-creation, ELSI governance, robust security, and early clinician workflow integration, the project aims to accelerate development of speech-based digital measures that are not only technically plausible but usable, trustworthy, and clinically meaningful.

## Data Availability

The original contributions presented in the study are included in the article/Supplementary Material, further inquiries can be directed to the corresponding author/s.
