# Supplementary material for: MSPEECH (multiple sclerosis monitoring through speech interaction in clinic and at home): a Living Lab study protocol for co-created, speech-based digital biomarkers in multiple sclerosis
Source: Front Digit Health. 2026 Jul 16;8:1830655. doi: 10.3389/fdgth.2026.1830655 (PMC13422476; doi:10.3389/fdgth.2026.1830655)
Supplement: Supplementary file 1 [file Datasheet1.pdf]

MSPEECH (Multiple Sclerosis Monitoring through Speech Interaction in Clinic and at Home): a Living Lab Study Protocol for Co-Created, Speech-Based Digital Biomarkers in Multiple Sclerosis

Tina Görbing*[1,2], Hernan Inojosa*[1,2], Anja Dillenseger[1,2], Johannes Tröger[3], Janna Hermann[3], Nicklas Linz[3], Stephen Gilbert[2,4], Tjalf Ziemssen[1,2]

[1]Center of Clinical Neuroscience, Department of Neurology, Faculty of Medicine and University Hospital Carl Gustav Carus, TUD Dresden University of Technology, Fetscherstraße 74, 01307 Dresden, Germany

[2]Centre for Tactile Internet with Human-in-the-Loop (CeTI), TUD Dresden University of Technology, Dresden, Germany

[3]Ki:Elements GmbH, Saarbrücken, Germany

[4]Else Kröner Fresenius Center for Digital Health, TUD Dresden University of Technology, Fetscherstraße 74, 01307 Dresden, Germany

*Shared first authorship

Corresponding author: Prof. Dr. Tjalf Ziemssen, Center of Clinical Neuroscience, Department of Neurology, Faculty of Medicine and University Hospital Carl Gustav Carus, TUD Dresden University of Technology, Fetscherstraße 74, 01307 Dresden, Germany, Email: tjalf.ziemssen@ukdd.de

Supplementary Table 1: Planned speech features, domains and exploratory clinical mapping in MSPEECH

Task Type	Planned Feature Domains	Example Features	Exploratory Clinical Domain
Picture description/free speech	Linguistic + fluency	speech rate, pause frequency, lexical diversity, utterance length	Cognition, fatigue, mood aspects
Verbal fluency	Semantic/phonemic retrieval	number of valid words, clustering, switching behavior	Cognition focus on executive function
Diary recall narration	Narrative organization	fluency, pauses, recall structure	Cognition focus on memory
Reading task	Acoustic + prosodic	articulation rate, pitch variability, intensity variation	Motor speech function, dysarthria, fatigue
Pa-Ta-Ka repetition	Articulatory motor control	syllable repetition rate, rhythm stability, timing variability	Motor speech function, dysarthria, articualtion
Sustained phonation	Voice quality	phonation duration, shimmer, jitter, harmonics-to-noise ratio	respiratory/voice function, voice quality
Dual-task speech + TDT	Cognitive-motor interference	speech timing under motor load, dual-task decrement	cognitive-motor integration, cognition and fine motor function

Footnote: Additional methodological specifications. Speech recordings will be stored as WAV files and harmonized before feature extraction where required. Planned analyses include acoustic, temporal, prosodic, articulatory, fluency-related and linguistic features. Acoustic and linguistic measures will be derived using established speech-processing and natural-language-processing pipelines; specific preprocessing parameters, including sampling-rate harmonization, frame segmentation, filtering and transcription procedures, will be documented before analysis for each demonstrator version. Because MSPEECH is an exploratory Living Lab protocol, these procedures define the planned analytical framework rather than a fixed biomarker pipeline.
